# Targeting miR-148b-5p Inhibits Immunity Microenvironment and Gastric Cancer Progression

**DOI:** 10.3389/fimmu.2021.590447

**Published:** 2021-02-24

**Authors:** Yuyu Zhang, Wei Huo, Lidi Sun, Jie Wu, Chengbin Zhang, Huanhuan Wang, Bin Wang, Jinlong Wei, Chao Qu, Hongshi Cao, Xin Jiang

**Affiliations:** ^1^ Department of Radiation Oncology, The First Hospital of Jilin University, Changchun, China; ^2^ Department of Pathology Oncology, The First Hospital of Jilin University, Changchun, China; ^3^ Department of Nursing, The First Hospital of Jilin University, Changchun, China

**Keywords:** gastric cancer, miR-148b-5p, ATPIF1, immune microenvironment, metabolic reprogramming

## Abstract

**Background:**

MicroRNAs (miRNAs) have been discovered to dictate the development of various tumors. However, studies on the roles of miRNAs in the progression of gastric cancer (GC) are still lacking.

**Methods:**

Herein, by analyzing GC cell lines and patients samples, we observed that miR-148b-5p was significantly downregulated in GC. We also confirmed that miR-148b-5p overexpression significantly inhibited GC cell proliferation and invasion *in vitro* and *in vivo*.

**Results:**

Overexpression of miR-148b-5p not only reprogrammed the metabolic properties of GC but also regulated the immune microenvironment by shifting lymphocyte and myeloid populations. Mechanistically, ATPIF1, an important glycolysis-associated gene, was identified as a direct target of miR-148b-5p and mediated the effect of miR-148b-5p. Notably, the low level of miR-148b-5p was significantly related with poor prognosis of GC patients (*P* < 0.001). Importantly, the levels of miR-148b-5p significantly changed the sensitivity of GC cells to several anti-cancer drugs (Doxorubicin, *P* < 0.05, Paclitaxel, *P* < 0.01, Docetaxel, *P* < 0.05).

**Conclusions:**

Targeting miR-148b-5p inhibits immunity microenvironment and gastric cancer progression.

## Background

Gastric cancer (GC) is the fifth most common cancer and the third most common cause of cancer death in the world (1). Even though its overall mortality rate significantly reduced in the last decade, GC has been shown to be still the third primary cause of tumor-associated death with approximately 720,000 deaths annually (2). In order to decrease the incidence and mortality of GC, improve the techniques of diagnosis and staging, and develop efficient therapeutic methods, the most important thing we first need to do is to further elucidate the underlying mechanisms of the carcinogenesis of GC.

The mitochondrial F1Fo-ATPsynthase catalyzes the synthesis of cellular ATP and is the master of oxidative phosphorylation (OXPHOS) (3). The ATPase inhibitory factor 1 (ATPIF1) is the most characterized biological inhibitor of the F1Fo-ATPsynthase (4). We previously reported that ATPIF1 upregulation resulted in an increased aerobic glycolysis in hepatocellular carcinoma (HCC) cells (4). By contrast, inhibition of ATPIF1 promoted OXPHOS due to reversal of the F1F0-ATP synthase, thus inhibiting HCC development (4). Thus, the control of ATPIF1 expression and activity is crucial as a strategy for producing novel agents to inhibit cancer development. However, very little is known about its role and regulation in GC.

MicroRNAs (miRNAs) are a group of endogenously short (about 17–26 nucleotides in length) conserved non-coding RNAs (5). *Via* binding to the 3′untranslated region (3′UTR) of their target gene mRNA, miRNA inhibits protein expression (6). Many literatures have summarized the roles of miRNAs in almost all pathological and physiological and pathological conditions, including the progression of tumors (7). Intriguingly, up to the difference of genes they target, miRNAs can switch from oncogenes to tumor suppressors (8). Recently, miR-148b-5p was suggested to play a critical role in tumor (9). However, the role, action mechanism, and potential significance of this miRNA in GC treatment are not clearly elucidated.

In this study, we aimed to reveal the effects of miR-148b-5p on GC cell proliferation, migration, metabolism, and immune microenvironment which remain unclear so far. Our new findings about the effects of miR-148b-5p on GC progression will certainly be helpful to elucidate and confirm several promising therapeutic targets for GC.

## Methods

### Cell Culture, Cell Counting, and Reagents

GES-1, NCI-N87, MKN28, SGC7901, AGS, MKN45, MGC803, and KATOIII cell lines were collected from Rutgers University. Almost all of these cells were cultured in Hyclone™ RPMI-1640 media (Invitrogen) containing 10% fetal bovine serum (FBS) except SGC7901, and GES-1 cells were cultured in Dulbecco’s modified Eagles medium (DMEM, Invitrogen) containing 10% FBS and KATOIII was supplemented with 20% FBS. All mediums were added with 1% penicillin/streptomycin (Invitrogen). These cells were grown in a humidified incubator under 5% CO_2_ at 37°C. Doxorubicin, Paditaxel, and Docetaxel were obtained from Sigma. Indicated GC cells were plated at 5.5 × 10^4^ and 1.5 × 10^5^ cells per well within 12-well plates, respectively. 16 h later, the indicated reagents were added, and cells were grown for 4 days, and then cell numbers were counted.

### Oligonucleotide, Plasmid Transfection, shRNA, and Bioinformatics Analysis

To figure out the possible downstream target genes of miR-148b-5p, three online software programs such as miRDB, TargetScan, and miRNA.org were used to predict the possible target genes of miR-148b-5p. MiR-148b-5p, miR-148b-5p inhibitor, short interfering RNA (siRNA) against ATPIF1 and negative control were purchased from Sigma and OriGene Technologies, respectively. Lipofectamine 3000 (Thermo Fisher Scientific) was used to transfect control vector and vector without the 3′-UTR of ATPIF1 or other plasmids in accordance with the manufacturer’s instructions. The lentiviral vectors were transfected into GC cells with a multiplicity of infection (MOI) of 40 to 50 in the presence of polybrene (5 μg/ml). At 48 h after transfection, stable cell lines were generated after puromycin (Sigma) selection (2 μg/ml), and gene silencing was verified by RT-qPCR and Western blot analysis. Then these cells were collected to perform western blot analysis assays and other biological assays. The ATPIF1 targeting sequences for the successfully knocking down ATPIF1 expression are:

5′-GGCGCTGGCTCCATCCGAGAAGCTGGTGG-3′ (cat#TG709527B) and 5′-ACTCGTCGGAGAGCATGGATTCGGGCGCT-3′ (cat# TG709527C).

### ELISA

The supernatants of indicated cell culture were collected, and the levels of TNFα, IL6, and CSF1 were quantified using Qauantikine ELISA Kit (R&D Systems).

### Profiling of Immune Cells

The cells from the indicated tumor tissue were isolated as described previously with some modifications (10). Briefly, single-cell suspensions were pre-incubated with anti-Fc receptor antibodies (BD Biosciences), stained for 30 min at 4°C, washed twice, and analyzed by flow cytometry to profile all immune cells. The anti-mouse antibodies used for flow cytometry were as follows: Gr-1 (RB6-8C5), CD45 (30-F11), TCRβ+CD11b (M1/70), F4/80 (BM8), CD11c (N418), (H57-597), CD8 (53-6.7), NK1.1 (PK136), Foxp3 (MF-14), (N418), Ly6G (1A8), CD206 (CD68C2), CD4 (RM4-5), and Ly6C (HK1.4).

### Oxygen Consumption Rate and Extracellular Acidification Rate Measurements

Cells were plated in XF24 cell culture microplates, and ECAR and OCR were assessed using an XF24 Analyzer (Seahorse Bioscience) as described (7). Briefly, the indicated cells were plated into the polystyrene cell culture plates of XF24 at 30,000/well. After incubation for 24 h with DMEM medium in a humidified 37°C incubator with 10% CO_2_, trypsin was added, and the cell number in each well was determined. OCR (pmoles/min) and ECAR (mpH/min) were reported as absolute rates or indicated as a percentage of the baseline oxygen consumption and normalized against cell counts. Each result was shown in triplicate minimally. And all results were normalized according to the total protein contents. The Bradford Protein Analysis reagents (Thermo Fisher Scientific) were used to measure the protein levels.

### Quantitative Reverse Transcription-Polymerase Chain Reaction

QRT-PCR was carried out according to previous publications (8). Briefly, RNA extraction was performed with TRIzol (Invitrogen) according to the manufacturer’s protocol. Ultraviolet spectrophotometry was used to determine the concentration and purity of RNA. A TaqMan Reverse Transcription Kit (Applied Biosystem) was used to perform reverse transcription (RT). ATaqMan miRNA Assay (Applied Biosystem) was used to perform qRT-PCR such as measuring the expression of miR-148b-5p gene. A SuperScript III One-Step RT-PCR kit (Applied Biosystem) was performed to examine ATPIF1 gene expression. The RT-PCR reaction mixture (20 µl) was subjected to 38 cycles: 30 s at 96°C, 30 s at 58°C, and 30 s at 72°C. For normalization, GAPDH, or U6 snRNA, and 18Ss RNA were used. The 2−ΔΔCt methods were used to analyze relative levels of gene expression. TNFα, IL6, ATPIF1, and CSF1 primers are as follows:

ATPIF1:5′-TTCGGTGTCGGGGTATGAAG-3′ and 5′-GCCCGTATCCATGCTATCCG-3′;

GAPDH:5′GACAAGCTTCCCGTTCTCAG-3′ and 5′-GAGTCAACGGATTTGGTCGT-3′;

IL6:5′-AGACAGCCACTCACCTCTTCAG-3′;5′-TTCTGCCAGTGCCTCTTTGCTG-3’;

TNFα: 5′-CTCTTCTGCCTGCTGCACTTTG-3′; 5′-ATGGGCTACAGGCTTGTCACTC-3′.

CSF1:5′-ATGGACACCTGAAGGTCCTG-3′;5′-GTTAGCATTGGGGGTGTTGT-3′.

U6:5′-GCTTCGGCAGCACATATACT-3′; 5′-GGTGCAGGGTCCGAGGTATT-3′; and 18S:5′-CCATCCAATCGGTAGTAGCG-3′and 5′-GTAACCCGTTGAACCCCATT-3′.

### Assays of Luciferase Reporter

Wild type (WT) or ATPIF1 3′-UTR mutant with or without putative miR-148b-5p seed sequence was subcloned into the psiCHECK-2™construct (Promega). Then these reporters were transfected into indicated GC cells along with either miR-148b-5p mimic or control. Lipofectamine 3000 reagent was used for transfection. 48 h later, the Dual Luciferase Reporter Assay System was used to analyze the luciferase activity as described previously (6).

### Performing Western Blotting

Ice-cold lysis buffer (4% sodium dodecyl sulfate, 0.1M Tris, pH 6.8, and 0.2 M dithiothreitol, and 20% glycerol) was used to collect the indicated cells or tissue samples. Then cell lysates were loaded onto 8–12% SDS-polyacrylamide gels, which was electrophoretically transferred onto PVDF membrane (Sigma). After blocking for 1 h using 10% non-fat milk in Tris-buffered saline with 1‰ Tween-20 (TBST), specified primary antibody such as ATPIF1 (1:500) from Santa Cruz and *β*-Actin (1:1,000) from Sigma as a loading control, respectively, was incubated in TBST containing 5% non-fat milk for overnight. Then secondary horseradish peroxidase (HRP)-linked antibodies (Bio-Rad) (1:1,000) was added for 1 h. Enhanced chemiluminescent substrate (Pierce) was used to detect the indicated proteins. ImageQuant TL software (Nonlinear Dynamics limited) was used to assay relative band intensities.

### Xenograft Model of Mice GC

BALB/C nude mice (six-week old) were obtained from Nanjing Animal Center. Following the National Institute of Health Guide for the Care and Use of Laboratory Animals and approved by the Institutional Animal Care and Use Committee and the Affiliated Hospital Ethics Committee of Jilin University, we performed all animal experiments. Briefly, GC cells with the indicated lentivirus expression plasmid against miR-148b-5p or negative control (scramble) were subcutaneously injected into the flanks of nude mice. After seven weeks, the nude mice were killed through cervical dislocation, and the tumors were measured, and experiments such as western blots and IHC were performed as described previously (11).

### Mice Metastasis Model

Indicated GC cells were injected into tail veins of female NOD/SCID mice (7 weeks old, 2 × 10^6^ cells/mice). 8 weeks after inoculation, mice were sacrificed. The lungs and livers were collected and tumor nodules were counted. Hematoxylin and eosin (H&E) staining and immunohistochemistry with the above tissues were performed.

### Patient and Tissue Samples

Tissue samples with GC were obtained from the First Affiliated Hospital, Jilin University from 2015 to 2018. All tissue samples from GC patients were kept at −80°C. The included patients gave their informed consent. The whole project was approved by the Ethics Committee of the First Affiliated Hospital of Jilin University.

### Proliferation, 3D Cell Culture, Migration, and Invasion Assays

Cells were directly counted using a TC20 Automated Cell Counter (Bio-Rad). Cell proliferation was measured with the CellTiter-Glo Luminescent Cell viability Assay (Promega).

For 3D cell cultures, cells were seeded onto growth factor reduced Matrigel (Life science). Spheroid growth was monitored, and the dimensions were measured as described previously. Spheroids were stained using LIVE/DEAD Viability/Cytotoxicity Kit (Molecular Probes) for microscopic visualization.

Migration and invasion experiments were performed as described previously through using a Transwell inserts (8 µm pore, BD Falcon) with or without Matrigel (12). Briefly, the indicated cells were plated in top chambers. The lower chamber was put into with 500 µl of DMEM containing 10% FBS. After incubation for 48 h, a cotton swab was used to scrape off the cells in the upper chamber. 0.1% Crystal Violet was used to stain the fixed migrated cells. For quantification, the stained cells were extracted with 10% acetic acid, and the absorbance was determined at 570 mm.

### Immunohistochemistry

Briefly, citric acid was used to incubate tissue sections for antigen retrieval. Then the sections were incubated with 3% H_2_O_2_ for 15 min to block the endogenous peroxidase. The primary antibody such as CD11b (1:500) or ATPIF1 (1:500) was added into the sections and incubated for overnight at 4°C. After washing, the appropriate secondary antibody was added into the tissue sections and incubated for I h at room temperature. Then staining with 3,3-diaminobenzidine and counterstaining with hematoxylin were carried out as previously described (13). Normal rabbit/mouse IgG antibodies functioned as negative control.

### Statistical Analysis

GraphPad Prism was used for generating graphs and performing statistical tests. All statistics were calculated using SPSS software (version 17.0; SPSS Inc., USA). For 2 × 2 tables, we used the Fisher’s exact test. To compare the significance of two groups, two-way assay of variance analysis and Student’s t-test were used. In addition, two-tailed Student’s t-test was used to measure differences among groups. Most data represent the mean ± SD. A p <0.05 was considered significant statistically. *, **, or *** indicate P <0.05, P <0.01, P <0.001, respectively. All experiments were at least repeated three times.

## Results

### The Level of miR-148b-5p Is Significantly Decreased in GC and Associated With Poor Prognosis

**Figure 1:**
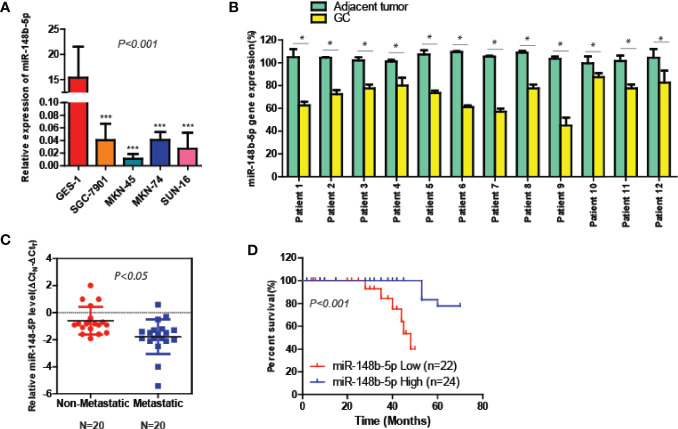
The level of miR-148-5p is significantly reduced in GC and tightly associated with poor prognosis. **(A)** The qRT-PCR analysis to measure the expression levels of miR-148b-5p in normal gastric epithelial cells and four GC cell lines. The result was normalized to the U6 expression. **(B)** The expression levels of miR-148b-5p in 12 pairs of GC patients. **(C)** The expression levels of miR-148b-5p in non-metastatic (N = 20) and metastatic tissues of GCs (N = 20) were analyzed by qRT-PCR. **(D)** Assays of Kaplan–Meier survival of 46 GC patients indicated that the lower expression of miR-148b-5p was associated with poor patient prognosis. *P < 0.05, ***P < 0.001.

To elucidate the roles of miR-148b-5p in GC, we first compared the expression levels of miR-148b-5p in the GC cell lines and normal stomach cells respectively. The qRT-PCR analysis indicated that compared with its expression in the normal gastric epithelial tissue, the level of miR-148b-5p was significantly decreased in all of GC cell lines (*P* < 0.001, **Figure 1A**). Additionally, we used qRT-PCR to analyze the paired GC tissues and non-tumor tissues in 12 patients. We found that the level of miR-148b-5p in tumor tissue was significantly lower than that in normal gastric epithelial tissue (*P* < 0.05, **Figure 1B**). It was also found that non-metastatic GC tissues had higher levels of miR-148b-5p when compared with metastatic GC tissues (*P* < 0.05, **Figure 1C**). Moreover, patients with lower level of miR-148b-5p exhibited shorter survival time, whereas the opposite results were observed with those with higher miR-148b-5p levels (*P* < 0.001, **Figure 1D**). Together, these findings strongly suggest that miR-148b-5p may be not only related to GC initiation and metastasis, but also taken as an importantly prognostic marker for GC patients.

### MiR-148b-5p Reprograms Metabolic Pathways and Inhibits GC Development

**Figure 2:**
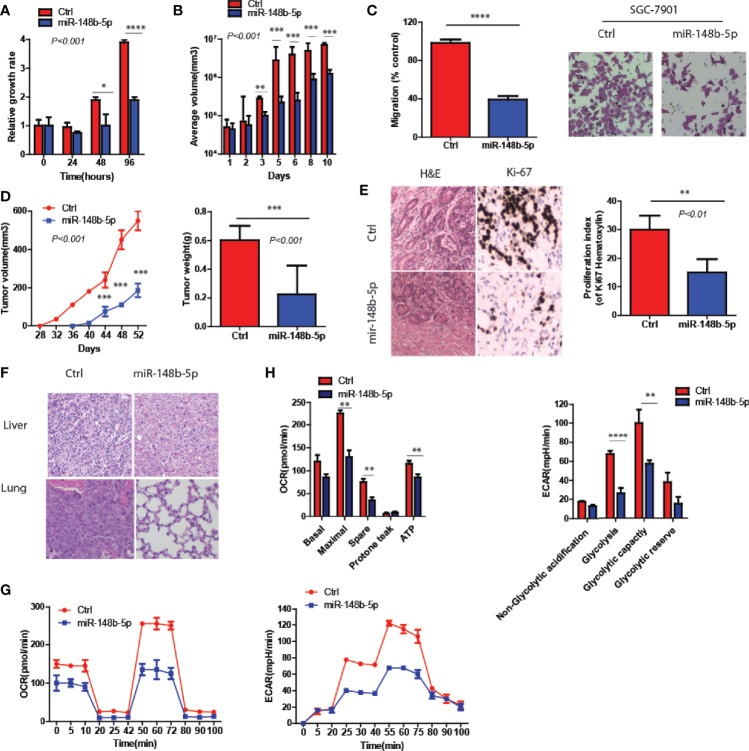
MiR-148b-5p reprograms metabolic pathways and inhibits GC development. **(A)** CellTiter -Glo methods and reagents were used to examine the effects of miR-148b-5p on GC cell growth. **(B)** The effect of miR-148b-5p on 3D spheroid growth at day 10. **(C)** The effect of miR-148b-5p on GC cell migration at 24 h. **(D)** The effect of miR-148b-5p on GC by injecting the indicated cells into the fat pads of nude mice (n = 10/group). **(E)** The representative images of H&E staining and Ki67 IHC from tumors. **(F)** H&E staining indicates liver and lung metastatic nodules from GC. **(G)** OCR assay was performed as indicated. **(H)** ECAR assay was performed as indicated. *P < 0.05, **P < 0.01, ***P < 0.001, ****P < 0.0001.

To clarify the function of miR-148b-5p in the development of GC, we first used GC cell lines to perform the gain- and loss-of-function experiments of miR-148b-5p, respectively. The data indicated that miR-148b-5p overexpression significantly reduced the growth rate and the size of 3D-cultured tumor spheroids (*P* < 0.001, **Figures 2A, B**). Also, high levels of miR-148b-5p significantly reduced the migratory activity of GC cells (*P* < 0.0001, **Figure 2C**).

*In vivo*, we performed the injection of GC cells transfected with control and miR-148b-5p respectively into the flanks of nude mice. We found that miR-148b-5p overexpression not only inhibited the xenograft tumor growth but also decreased the number of Ki67^+^ cells (*P* < 0.001, **Figures 2D, E**). Furthermore, as compared with those from cells overexpressing miR-148b-5p, the tumors of mice xenograft from control cells often developed liver or lung metastases (**Figure 2F**).

Since glycolysis and oxidative phosphorylation determined the development of GC (14), we also examine the metabolic characteristics associated with miR-148b-5p. We found that as compared to the control groups, overexpression of miR-148b-5p significantly downregulated oxygen consumption rate (OCR) as well as extracellular acidification rate (ECAR) (**Figures 2G, H**).

Together, these data indicate that miR-148b-5p significantly inhibits GC growth and metastasis *in vitro* and *in vivo*.

### ATPIF1 Is Identified as a Downstream Target of miR-148b-5p in GC Cells

**Figure 3:**
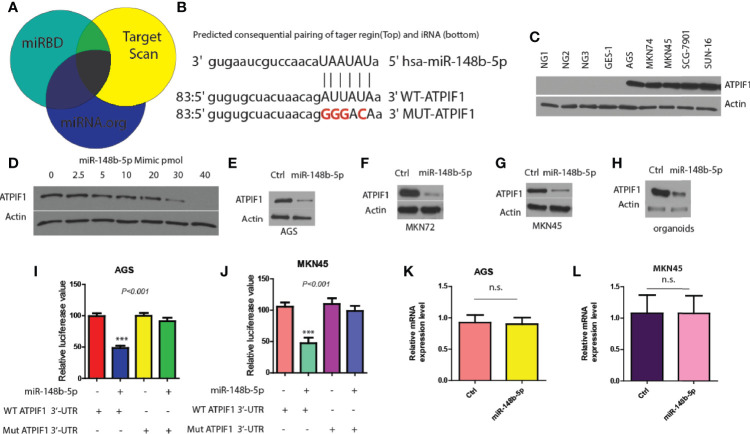
ATPIF1 is identified as a downstream target of miR-148b-5p in GC cells. **(A)** Target gene of miR-148b-5p by predicted using three online databases. **(B)** One predicted binding motif of miR-148b-5p on the 3′-UTR of ATPIF1 was presented. **(C)** Western blot presented ATPIF1 protein expression levels in five GC cell lines (AGS, SGC-7901, MKN74, MKN45, and SNU-16) and four normal human gastric cells. **(D)** Western blot data indicated the effect of transient reconstitution of miR-148b-5p using a mimic (0–40 pmol) on the expression of ATPIF1 in SCG-7901 cells. The effects of reconstitution of miR-148b-5p on the expression of ATPIF1 in AGS cells **(E)**, MKN74 cells **(F)**, MKN45 cells **(G)**, and mice GC organoids **(H)**. The effects of of miR-148b-5p on WT or mutant ATPIF1-3′-UTR luciferase reporter in AGS **(I)** or MKN45 cells **(J–L)** The effects of miR-148b-5p on ATPIF1 mRNA gene expression level using qRT-PCR analysis in AGS and MKN45 cells. ***P < 0.001; n. s., no statistical difference.

Next, three miRNA databases (Target Scan, miRBD, and miRNA.org) were used to predict common downstream targets of miR-148b-5p in GC (15). It was found that ATPIF1, a mitochondrial ATPase inhibitor, might be a predicted target gene (**Figure 3A**). And analysis predicted a possible miR-148b-5p binding element within the 3′-untranslated region (3′-UTR) of ATPIF1 (**Figure 3B**). To test this hypothesis, we determined the effect of miR-148b-5p on the protein level of ATPIF1 in GC cells. Western blot data showed that the expression of ATPIF1 was remarkably higher in several GC cell lines than in normal control cell lines (**Figure 3C**). However, reconstitution of miR-148b-5p using an increasing-mimic significantly decreased the levels of ATPIF1 protein, while there was no notable change in the levels of beta-actin protein in SGC-7901 cells, indicating that ATPIF1 is a specific downstream target of miR-148b-5p (**Figure 3D**). Western blot analysis in AGS, MKN72, and MKN45 cells also indicated similar observation (**Figures 3E–G**). Furthermore, miR-148b-5p reconstitution significantly reduced the levels of ATPIF1 protein in dysplastic gastric organoids, as compared with controls (**Figure 3H**).

In addition, we found that the mutation of ATPIF1 3′-UTR (lacking miR-148b-5p binding site) did not significantly affect luciferase reporter activity, while WT-ATPIF1 3′-UTR reporter luciferase value was decreased in these in AGS and MKN45 cells compared with control cells (*P* < 0.001, **Figures 3I, J**). Moreover, the significant differences in the levels of ATPIF1 mRNA were not observed after reconstitution of miR-148b-5p in MKN45 or AGS cells, compared with control cells (**Figures 3K, L**). These data suggest that miR-148b-5p directly binds to the 3′-UTR of ATPIF1, thereby reducing its protein levels in GC cells.

### Restoration of ATPIF1 Significantly Rescues the Effects of miR-148b-5p on GC Progression

**Figure 4:**
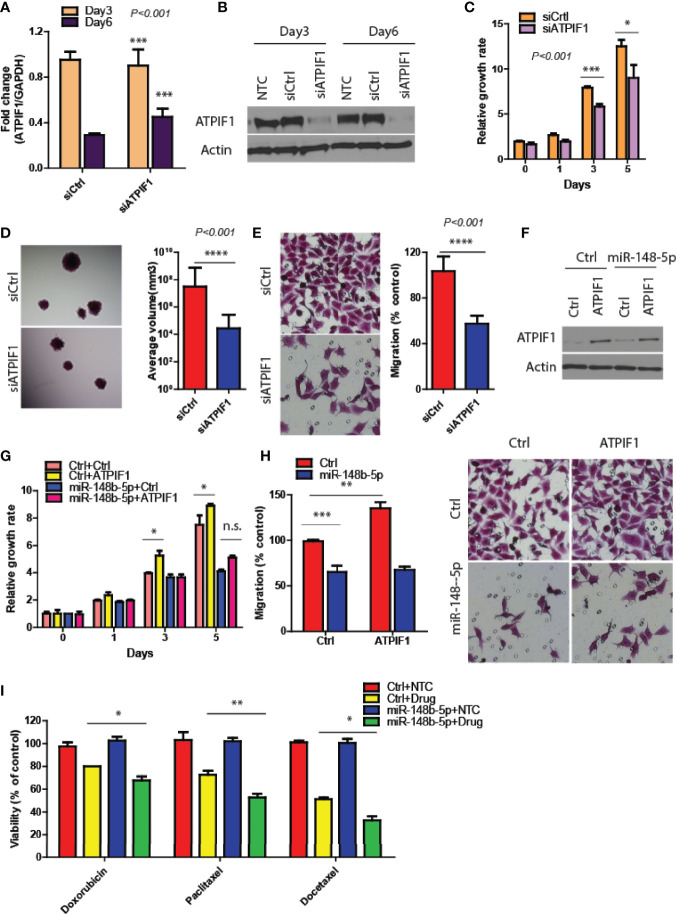
Restoration of ATPIF1 significantly rescues the effects of miR-148b-5p on GC progression. **(A, B)** The levels of ATPIF1 mRNA and protein after siRNA treatment. Three days after transfection, GC cell proliferation **(C)**, 3D tumor growth **(D)** and migration assay **(E)** were examined respectively. **(F)** Western blots of ATIF1 levels were performed. And the proliferation **(G)** and migration assays **(H)** were conducted. **(I)** The effects of Paclitaxel, (100 nmol/L), Doxorubicin (0.3 mmol/L), and Docetaxel (10 nmol/L) on the cell viability of GC cells were examined after hours. The relative growth rate or viability after drug treatment was shown. *P < 0.05, **P < 0.01, ***P < 0.001, ****P < 0.001; n. s., no statistical difference.

To verify that ATPIF1 mediated the downstream effects of miR-148b5p in GC cells, we first downregulated ATPIF1 level in MKN45 cells using siRNA (*P* < 0.001, **Figures 4A, B**), which results in the inhibition of GC cell proliferation, 3D tumor growth, and migratory activity (*P* < 0.001, **Figures 4C–E**). In contrast, ATPIF1 overexpression resulted in increased cell proliferation and migration in SCG-7901 cancer cells (data not shown). However, overexpression of ATPIF1 in miR-148b-5p–overexpressing SGC-7901 cells failed to induce such changes, indicating that the inhibitory effect of miR-148b-5p on GC cells depends on the protein levels of ATPIF1 (**Figures 4F–H**). Given that ATPIF1-mediated metabolic pathways such as glycolysis and OXPHOS had been reported to be widely involved in chemotherapeutic resistance (4), we examined the possible response to three chemotherapeutic agents in GC cells with miR-148b-5 overexpression. We found that miR-148b-5p overexpression significantly increased sensitivity to Doxorubicin (*P* < 0.05), Paditaxel (*P* < 0.001), and Docetaxel (*P* < 0.05) as compared with control cells (**Figure 4I**). These findings demonstrate that ATPIF1 is a key downstream effector of miR-148b-5p and that miR148b-5p transfection sensitizes GC cells to several cytotoxic chemotherapeutic agents.

### MiR-148b-5p Overexpression Reprograms GC Immune Microenvironment

**Figure 5:**
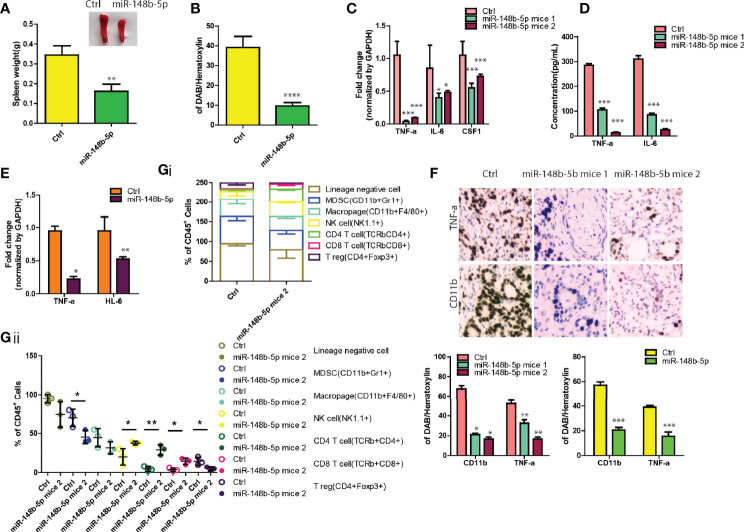
MiR-148b-5p overexpression reprograms GC immune microenvironment. **(A)** Representative spleen pictures were presented and weight was measured from BALB/c nude mice xenograft bearing indicated GC cells. **(B)** Spleens tissues were stained by using CD11b antibody and its percentage was shown. **(C)** The levels of TNFa, IL-6, and CSF1 mRNA were analyzed using qRT-PCR in GC tissues of mice bearing mir-148b-5p overexpressing-MKN45 cells or not. **(D)** Secreted TNFα, IL-6, and CSF1 in GC cells were measured. **(E)** The levels of TNFα, IL-6, and CSF1 mRNA were analyzed using qRT-PCR in mice bearing mir-148b-5p overexpressing-AGS cells or not. **(F)** IHC staining were performed with TNFα and CD11b antibodies in GC tissues of mice bearing miR-148b-5p overexpressing-MKN45 cells or not. **(G)** Profiling of immune cell populations in in GC tissues of mice bearing miR-148b-5p overexpressing-MKN45 cells or not were performed using FACS analysis. *P < 0.05; **P < 0.01; ***P < 0.001, ****P < 0.001.

Since the immunity microenvironment affects the progression of GC (16), we then performed the *in vivo* xenograft experiments. As shown by **Figure 5A**, the spleens of the mice injected with control-overexpressing GC cells indicated spleen enlargement while mice bearing miR-148b-5p-overexpressing tumors had significantly reduced spleen volumes (*P* < 0.01). Consistent with these findings, mice bearing the miR-148b-5p-overexpressing tumors were observed having a marked decrease of splenic CD11b^+^ myeloid cells as compared with the control group (*P* < 0.0001, **Figure 5B**). Furthermore, we found that several immune-related genes within GC tissues such as TNFa, IL-6, and Csf1 showed significantly negative association with the miR-148b-5p levels (**Figure 5C**). Downregulation of these cytokines at protein levels significantly decreased and was observed in tumor tissues (**Figure 5D**). The downregulation of above cytokines in the cells overexpressing miR-148b-5p were validated in AGS cells (**Figure 5E**). Meanwhile, the infiltration of CD11b^+^ myeloid cells was significantly reduced in cancers with miR-148b-5p-overexpressing GC cells (**Figure 5F**).

To more comprehensively examine the effects of the miR-148b-5p on the reprogramming of GC immune microenvironment, we collected the allograft tumors with miR-148b-5p overexpression in a syngeneic mouse GC model and used polychromatic flow cytometry to perform immune profiling. As compared with the control tumors, the miR-148b-5p-overexpressing GC tissues indicated the significant reductions in myeloid-derived suppressor cells (MDSCs), macrophages, and T regulatory cells in the miR-148b-5p-overexpressing GC tissues whereas the NK cells, CD4^+^ T cells, and CD8^+^ T cells were significantly increased (**Figures 5Gi, ii**), suggesting the effects of miR-148b-5p on the composition of various immune cells in the tumor microenvironment. Therefore, our data suggest that miR-148b-5p might promote GC growth and metastasis through reprogramming GC immune microenvironment beyond its role in metabolism.

## Discussion

Dysregulation of miRNAs was known to play important roles in the pathological processes of various tumors, including GC (17). By analyzing the expression of miRNAs in GC patient samples and cell lines, we found that miR-148b-5p expression was downregulated in GC tissues and GC cell lines as compared to the controls, and that low levels of miR-148b-5p were related to poor prognosis for patients with GC. Loss-of-function experiments confirmed that the depletion of miR-148b-5p markedly increased the proliferation and invasion abilities of GC cells. *In vivo*, mice xenograft model demonstrated an inhibitory ability of miR-148b-5p in GC development. Furthermore, the infiltrating population of MDSC and macrophage and GC metabolism reprogramming were positively correlated with miR-148b-5p deficiency. Then we indicated that miR-148b-5p targeted ATPIF1 and inhibited GC cell proliferation. Importantly, a miR-148b-5p mimic or ATPIF1 inhibitor upregulated the therapeutic efficacy of anti-GC drugs in a subgroup of GC patients. Collectively, our results demonstrate that miR-148b-5p deficiency-mediated development of GC is partially attributed to the aberrant reprogramming in metabolism and tumor immunity microenvironment in GC, and that the miR-148b-5p/ATPIF1/TNFa plus IL6 and CSF1 axis establishes the groundwork to further develop more-personalized therapeutic methods for GC patients.

Human epidermal growth of receptor 2 (HER2, p185) overexpression has been shown to significantly contribute to the proliferation and survival of cancer cells (18). Although in GC, HER2 is not frequently expressed, clinicopathologically, its levels have been demonstrated to correlate strongly with GC types (19). Data provided herein elucidated that the downregulation of miR-148b-5p was mainly observed in GC cell lines (AGS, MKN45, MKN74, MGC803, and SGC7901 cell lines), which lack the HER2 protein. However, this change of miR-148b-5p was not observed in HER2-positive cell lines such as NCI-N87, MKN-7 and KATO-III (data not shown) (20). The similar findings were confirmed in the clinical samples. Therefore, we further investigated the effect of miR-148b-5p on HER2-negative GC cells. For the first time, our results indicate that miR-148b-5p acts as a tumor suppressor microRNA in HER2-negative GC cells. Moreover, we further identified ATPIF1 as a novel critical downstream effector of miR-148b-5p in GC even though one previous study demonstrated miR-29 regulating ATPIF1 level in breast cancer (21). In future study, we need to examine whether or not miR-148b-5p has similar effects on the HER2-positive GC cells.

This study also reveals another interesting finding that the miR-148b-5p reprograms the immune microenvironment in GC. Although most previous studies about tumor microenvironment primarily focused on molecules, cytokines, or other proteins secreted by cancer cells or immunological cells, the roles of miRNAs have recently gained increasing attention and greater emphasis (22). That is because the bidirectional infiltration of miRNAs between tumor cells and stromal cells has been shown playing critical roles and taken as a key factor in cancer progression (23). For example, the aberrant miRNA levels dictated the imbalance of Treg/Th17 cell ratio in epithelial ovarian cancer (24). The colon cancer cell migration and invasion were mainly ascribed to the aberrant miR-155 and miR-21 levels contained in the exosomes (25). Consistent with these previous findings, in this study, we identified a novel miR-148b-5p as a critical regulator of tumor immunity environment.

In addition, our data provided direct evidence which further reinforces the notion that tumor metabolism and cytokine-related pathway are associated with tumor immune inhibition (26). For the first time, we demonstrated that GCs with miR-148b-5p deficiency had not only lower levels of TNFa, IL6, and CSF1 but also less infiltration of CD8^+^T and NK cells than the control GCs. Moreover, we also observed a negative relationship between miR-148b-5p and MDSC cells while a positive relationship between TNFa or ATPIF1 and CD11b in GC tissues. All these findings reveal a close relationship between the miR-148b-5p/ATPIF1 axis and the tumor immunosuppressive microenvironment. In future study, we need to further determine that the administration of a miR-148b-5p mimics or ATPIF1 inhibitor could function with anti-PD1 treatment synergistically in immunocompetent GC models. Simultaneously, TNFa, IL6, and CSF1 were downregulated by the miR-148b-5p mimic administration. Thus, we can indicate that the miR-148b-5p/ATPIF1/TNFa plus IL6 and CSF1 axis is a vital mechanism regulating GC immunosuppression, suggesting that a subgroup of patients might benefit from miR-148b-5p mimics or ATPIF1 inhibitor administration.

Notably, our current study still has several limitations to be taken into account. Although a prominent difference in miR-148b-5p expression was demonstrated between GCs and the controls, we did not clarify the profiles of whole cellular miRNA in GCs. Another limitation is that our analysis on the mouse microenvironment in GC xenograft tumors might not fully represent the human microenvironment because we used severely immunocompromised mice. Thirdly, we did not describe one more comprehensive profiling of tumor microenvironment related to the different levels of miR-148b-5p. Further characterization of immune microenvironment in response to miR-148b-5p including characterization of diverse immune cell infiltration is needed to obtain more clear roles of miR-148b-5p in GC progression. Additionally, although our data identified ATPIF1 as a downstream effector, we cannot rule out miR-148b-5p possibly functioning through other target genes or other signal pathways. Therefore, to provide a rationale for therapies, the identification and verification of other targets, and their effects, are imperative.

In summary, this study provides the first evidence that miR-148b-5p acts as a tumor suppressor miRNA, and that miR-148b-5p deficiency induces GC development and immune tolerance *via* the miR-148b-5p/ATPIF1/TNFa plus IL6 and CSF1 axis. Importantly, a miR-148b-5p mimic or ATPIF1 inhibitor was shown to promote the efficacy of anti-GC drug treatment in a subgroup of GC patients.

## Conclusions

These findings reveal previously unrecognized roles of tumor suppressor miR-148b-5p in GC development, suggesting the miR-148b-5p/ATPIF1 axis as a therapeutic target and potential prognostic biomarker for patients with GC.

## Data Availability Statement

The raw data supporting the conclusions of this article will be made available by the authors, without undue reservation.

## Ethics Statement

The studies involving human participants were reviewed and approved by The Medical College Committee of the First Hospital of Jilin University. The patients/participants provided their written informed consent to participate in this study. The animal study was reviewed and approved by Animal ethics committee of the first hospital of Jilin University Animal experiment ethics.

## Author Contributions

YZ, WH, and LS: study design, acquisition of data, analysis, and interpretation. JW, CZ, and HW: acquisition of data and technical support. BW and JLW: drafting of the manuscript and material support. CQ, HC, and XJ: study design, drafting, and critical revision of the manuscript and obtaining funding. All authors contributed to the article and approved the submitted version.

## Funding 

This study was supported by the National Natural Science Foundation of China (No. 81702744). The funders had no role in study design, data collection and analysis, decision to publish, or preparation of the manuscript.

## Conflict of Interests

The authors declare that the research was conducted in the absence of any commercial or financial relationships that could be construed as a potential conflict of interest.

## Abbreviations

OXPHOS, oxidative phosphorylation; ATPIF1, ATPase inhibitory factor 1; HCC, hepatocellular carcinoma; GC, gastric cancer; TNFa, tumor necrosis factor α; IL-6, Interleukin 6; Csf1, colony stimulating factor 1.
